# Coral Hydrate, a Novel Antioxidant, Improves Alcohol Intoxication in Mice

**DOI:** 10.3390/antiox11071290

**Published:** 2022-06-29

**Authors:** Hung-Tsung Wu, Ting-Hsing Chao, Horng-Yih Ou, Liang-Miin Tsai

**Affiliations:** 1 Department of Internal Medicine, School of Medicine, College of Medicine, National Cheng Kung University, Tainan 701, Taiwan; z11008014@ncku.edu.tw (H.-T.W.); chaoth@mail.ncku.edu.tw (T.-H.C.); wahoryi@mail.ncku.edu.tw (H.-Y.O.); 2 Department of Internal Medicine, National Cheng Kung University Hospital, College of Medicine, National Cheng Kung University, Tainan 701, Taiwan; 3 Department of Internal Medicine, Tainan Municipal Hospital (Managed by Show-Chwan Medical Care Corporation), Tainan 701, Taiwan

**Keywords:** alcohol, alcohol dehydrogenase, catalase, coral hydrate, hangover, intoxication

## Abstract

Alcohol-drinking culture may cause individuals to periodically experience unpleasant hangovers. In addition, ethanol catabolism stimulates the production of free radicals that may cause liver injury and further lead to the development of chronic alcoholic fatty liver disease. Although a number of studies have suggested that hydrogenated water may be consumed to act as free radical scavenger, its instability limits its application. In this study, we used coral hydrate (i.e., hydrogenated coral materials) as a more stable hydrogen source and evaluated its effects in a murine model of alcohol intoxication. In solution, coral hydrate exhibited much more stable redox potential than did hydrogenated water. Furthermore, administration of coral hydrate by oral gavage significantly prolonged the time to fall asleep and decreased the total sleep time in mice that received intraperitoneal injection of ethanol. The mice receiving coral hydrate also had lower plasma ethanol and acetaldehyde levels than controls. In line with this observation, hepatic expression of alcohol dehydrogenase, acetaldehyde dehydrogenase, catalase and glutathione peroxidase were all significantly increased by the treatment. Meanwhile, alcohol-induced upregulation of pro-inflammatory factors was attenuated by the administration of coral hydrate. Taken together, our data suggest that coral hydrate might be an effective novel treatment for alcohol intoxication.

## 1. Introduction

Drinking culture is a worldwide phenomenon, but long-term alcohol consumption may lead to alcoholic fatty liver disease [1]. In addition to increasing the risk of chronic disease, excessive ethanol intake can cause acute unpleasant physiological and psychological effects, including hangover [2]. Hangover may involve headache, feelings of dizziness or fainting, tiredness, and also gastrointestinal distress or sleep pattern changes [3].

Alcohol metabolism occurs mainly in the liver, where ethanol is first converted to acetaldehyde by alcohol dehydrogenase (ADH) and then to acetic acid by acetaldehyde dehydrogenase (ALDH) [4]. Acetaldehyde is a toxic metabolite that can directly react with biomolecules or lead to the production of reactive oxygen species (ROS) [5]. It has been proposed that alcohol hangover largely results from the accumulation of acetaldehyde and consequent decrease of antioxidant protection systems [6,7]. In addition, acetaldehyde-mediated induction of pro-inflammatory cytokines has been shown to play a role in generating hangover symptoms [8]. Although many of the mechanisms responsible for hangover are known, effective treatments and preventative agents for alcoholic intoxication are still scarce.

Hydrogenated water is made by saturating regular water with hydrogen gas, and the product is generally recognized as safe by the United States Food and Drug Administration [9]. In a randomized controlled pilot trial, hydrogenated water was found to reduce liver fat accumulation and improve liver enzyme profiles in patients with non-alcoholic fatty liver disease [10]. Furthermore, dissolving hydrogen in haemodialysis solutions can decrease plasma concentrations of pro-inflammatory factors in haemodialysis patients [11]. Intake of hydrogenated water prior to exercise was also reported to reduce fatigue [12]. Although numerous studies have illustrated the beneficial effects of hydrogenated water in human health, the instability of its redox status and difficulties in quantifying the levels of dissolved hydrogen in water have limited its application. These challenges have also prevented the establishment of optimal intake protocols [13]. Moreover, the effects of dissolved hydrogen on alcoholic intoxication or hangover remain unknown.

In this study, coral hydrate, utilizing a porous coral material as a stabilizing agent for chelated hydrogen, was used. This reagent was first compared with hydrogenated water in terms of redox potential stability. Then, the effects of coral hydrate were evaluated in a mouse model of alcohol intoxication.

## 2. Material and Methods

### 2.1. Redox Potential of Coral Hydrate

Porous coral materials with or without hydrogenation were purchased from HoHo Biotech Co., Ltd. (Taipei, Taiwan). To generate hydrogenated water, hydrogen gas (99.9%, Yun-Shan, Tainan, Taiwan) was bubbled directly into Milli-Q water at 1 L/min for 10 min. The hydrogen content was 1200 ppb, pH 7.15, and Eh −556 mV. Coral hydrate (100 mg) was suspended in 10 mL Milli-Q water, and the redox potentials of coral hydrate and hydrogenated water were evaluated over time using an oxidation reduction potential electrode (Dogger company, Taipei, Taiwan).

### 2.2. Animal Treatments

All animal experiments were approved by the Institutional Animal Care and Use Committee of National Cheng Kung University (IACUC No: 110-308) and performed in accordance with National Institutes of Health guide for the care and use of laboratory animals. Eight-week-old C57BL/6 male mice were purchased from the National Laboratory Animal Center (Taipei, Taiwan) and housed in a temperature- and humidity-controlled room (23 ± 2 °C; 60 ± 0% RH). The mice were randomly divided into four groups: (1) mice treated with 420 mg/kg porous coral calcium dissolved in 3% carboxymethyl cellulose (Sigma-Aldrich, St. Louis, MO, USA) (Vehicle group); (2) mice treated with 105 mg/kg coral hydrate (Low-dose group); (3) mice treated with 210 mg/kg coral hydrate (Medium-dose group); and (4) mice treated with 420 mg/kg coral hydrate (High-dose group). The doses of coral hydrate used in the present study were selected based on the manufacturer’s suggestion for daily consumption; doses for mice were calculated using the relative body surface formula for dose translation described in a previous study [14]. Coral hydrate was administered to mice once daily by oral gavage for seven days before the intraperitoneal injection of 20% ethanol at a dose of 3.8 g/kg body weight, similar to the protocol described previously [15]. After the injection of ethanol, lack of righting reflex for 1 min was used as an index of falling into sleep, and the latency and duration of sleeping time were recorded. Before the injection of ethanol, the mice were subjected to overnight fasting, and the blood samples were collected for further experiments. Blood biochemistry analyses, including measurements of blood glucose, alanine transaminase (ALT), blood urea nitrogen (BUN), creatinine, triglyceride, total cholesterol, and high-density lipoprotein-cholesterol (HDL-C) were performed using an automated clinical chemistry analyzer (Fuji film, Tokyo, Japan). The estimated glomerular filtration rate (eGFR) was calculated using chronic kidney disease epidemiology collaboration equation. The plasma ethanol and acetaldehyde concentrations were determined using a commercial assay kit (Abcam, Cambridge, UK). Each group of mice was sacrificed 2 h after the injection of ethanol, and liver tissues were removed for further experiments.

### 2.3. Western Blots

After administration of coral hydrate for seven days, mice were well-anesthetized, and liver tissues were removed for Western blot analysis. The tissues were homogenized in RIPA buffer (Protech Technology, Taipei, Taiwan) with a protease inhibitor cocktail (Sigma-Aldrich). After centrifugation at 13,000 rpm for 10 min at 4 °C, the supernatant was collected, and protein concentrations were measured with the bicinchoninic acid assay (Visual Protein, Taipei, Taiwan).

Protein samples (30 μg) were separated by sodium dodecyl sulfate-polyacrylamide gel electrophoresis, and transferred to polyvinylidene difluoride membranes (Biomate, Kaohsiung, Taiwan). The membranes were blocked with 10% skim milk in Tris buffered-saline with 0.05% Tween 20 for 1 h at room temperature and then incubated with 1:1000 primary antibody against catalase (Cell Signaling, Danvers, MA, USA), manganese superoxide dismutase (MnSOD; Cell Signaling), glutathione peroxidase (GPx; Cell Signaling), alcohol dehydrogenase (ADH; Abcam) acetaldehyde dehydrogenase (ALDH; Abcam), or actin (Millipore, Temecula, CA, USA) at 4 °C overnight. The blots were then incubated with horseradish peroxidase-conjugated secondary antibodies at room temperature for 1 h. The bands were detected using enhanced chemiluminescence kit (Millipore), and the band densities were quantified using ImageJ software (https://imagej.nih.gov/nih-image/) (accessed on 1 October 2021).

### 2.4. Quantitative Polymerase Chain Reaction (qPCR)

Hepatic inflammation was evaluated by performing qPCR on a set of inflammation-related genes. Briefly, total RNA from the liver samples was isolated with Rezol^TM^ (Protech Technology). The RNA samples (2 μg) were reverse transcribed using a Moloney murine leukemia virus reverse transcription kit (Protech Technology). The primers used in this study were as follows: *Homo sapiens* CCL2 (NM_002982.4) forward 5′-CCCCAGTCACCTGCTGTTAT-3′, reverse 5′-TGGAATCCTGAACCCACTTC-3′; *Homo sapiens* TNFA (NM_000594.4) forward 5′-TCCTTCAGACACCCTCAACC-3′; reverse 5′-CACATTCCTGAATCCCAGGT-3′; *Homo sapiens* IL6 (NM_000600.5) forward 5′- CCTTCCAAAGATGGCTGAAA-3′; reverse 5′-GCTCTGGCTTGTTCCTCACT-3′. qPCR was performed using a StepOnePlus Real-Time PCR System according to the manufacturer’s instructions (Santa Clara, CA, USA). RNA expression levels were calculated by the 2^−ΔΔCt^ method, using Glyceraldehyde 3-phosphate dehydrogenase (GAPDH) as the internal standard.

### 2.5. Statistics

Data are expressed as mean ± standard error of mean (SEM). Statistical analyses were conducted using one-way analysis of variance (ANOVA), followed by Tukey’s post hoc test. *p* < 0.05 was considered statistically significant.

## 3. Results

### 3.1. Coral Hydrate Retains Redox Potential Longer Than Hydrogenated Water

**Figure 1:**
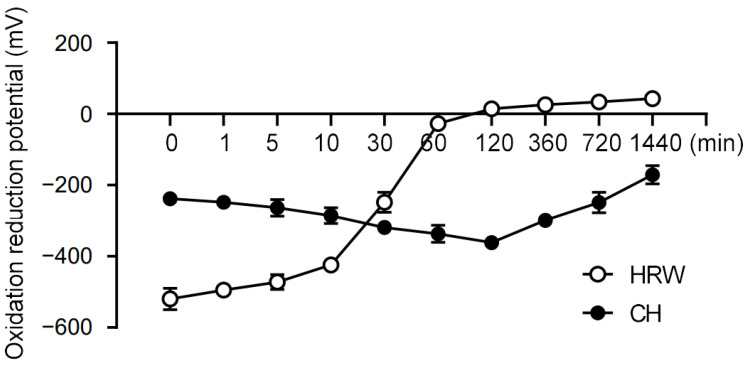
Coral hydrate has stable redox potential. The redox potentials of hydrogenated water (HRW), and coral hydrate (CH; 100 mg in 10 mL MQ) were measured using oxidation/reduction electrodes at the indicated times.

To begin our study, we compared the redox stability of coral hydrate with hydrogenated water. Notably, we found that the redox potential of hydrogenated water changed drastically within 1 h, whereas coral hydrate exhibited much better stability, sustaining its redox potential for at least 24 h (Figure 1). Based on this drastic improvement in stability, we decided to utilize coral hydrate treatments throughout our further experiments on hangovers.

### 3.2. Administration of Coral Hydrate Changes Sleep Pattern after Ethanol Injection

**Figure 2:**
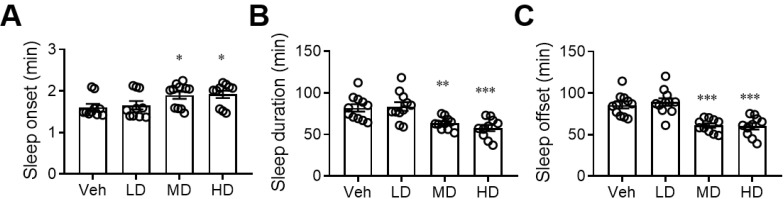
Sleep duration of ethanol-injected mice is diminished by coral hydrate treatment. C57BL/6 mice were treated by oral gavage with 105 mg/kg coral hydrate (Low dose group; LD), 210 mg/kg coral hydrate (Medium dose group, MD) or 420 mg/kg coral hydrate (High dose group; HD), and vehicle group (Veh), 420 mg/ kg coral calcium dissolved in 3% carboxymethyl cellulose. The coral hydrate was administered for seven days before intraperitoneal injection of ethanol. The time of sleep onset (**A**), sleep duration (**B**), and sleep offset (time to waking) (**C**) were recorded. N = 11 mice in each group; * *p* < 0.05; ** *p* < 0.01; *** *p* < 0.001 as compared with vehicle group.

After finding that coral hydrate has sufficient redox stability, we sought to evaluate its effects on alcohol intoxication. We found that daily administration of coral hydrate for 7 days not only prolonged the time it took for mice to fall asleep after ethanol injection (Figure 2A), but it also decreased the total time spent sleeping (Figure 2B). In addition, the time to waking was significantly decreased by administration of coral hydrate (Figure 2C), implying that the treatment might have potential to improve ethanol intoxication.

### 3.3. Coral Hydrate Decreases Blood Ethanol Concentrations and Increases Hepatic Levels of ADH and ALDH

**Figure 3:**
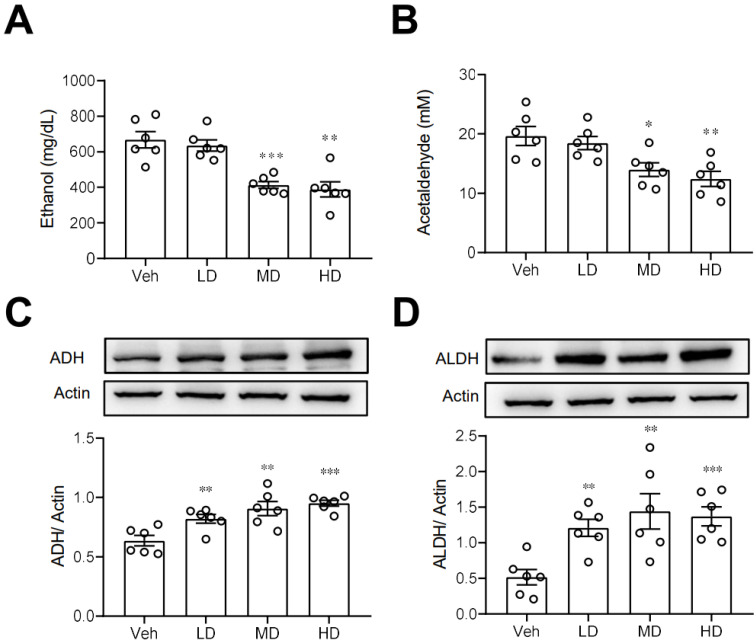
Ethanol catabolism is enhanced by coral hydrate in mice. Coral hydrate was administered to mice via oral gavage for 7 days before the intraperitoneal injection of ethanol. Each group of the mice were fasted overnight prior to ethanol injection. Blood samples were collected for the determination of plasma ethanol (**A**) and acetaldehyde (**B**) concentrations using a commercially available kit. Two hours after the injection of ethanol, experimental animals of each group were sacrificed, and liver tissues were removed for measurements of alcohol dehydrogenase (ADH) (**C**), and acetaldehyde dehydrogenase (ALDH) (**D**) by Western blot. Low-dose group (LD), 105 mg/kg coral hydrate; Medium-dose group (MD), 210 mg/kg coral hydrate; High-dose group (HD), 420 mg/kg coral hydrate; vehicle group (Veh), 420 mg/ kg coral calcium dissolved in 3% carboxymethyl cellulose. N = 6 mice for each group; * *p* < 0.05; ** *p* < 0.01; *** *p* < 0.001 as compared with vehicle group.

We next investigated some possible routes by which coral hydrate may improve ethanol intoxication or hangover. First, we measured the plasma concentrations of ethanol in mice treated with coral hydrate. As shown in Figure 3A,B, plasma ethanol and acetaldehyde concentrations were significantly decreased in coral hydrate-treated groups. Since ADH and ALDH are the main enzymes responsible for the catabolism of ethanol, we then examined the protein levels of ADH and ALDH in liver, and we found that both ADH (Figure 3C) and ALDH (Figure 3D) were significantly upregulated in the livers of mice treated with coral hydrate. Together these findings suggest that ethanol catabolism may be upregulated by the coral hydrate treatment.

### 3.4. Coral Hydrate Increases Levels of Hepatic Catalase and Glutathione Peroxidase

**Figure 4:**
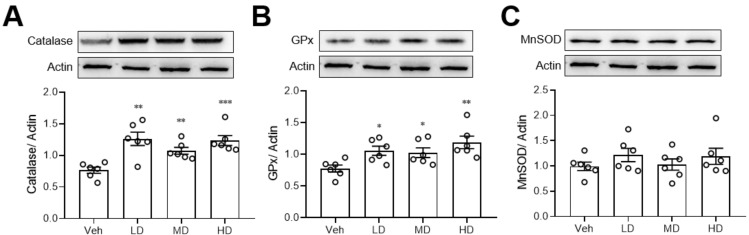
Coral hydrate increases anti-oxidative enzyme levels in ethanol-injected mice. Coral hydrate was administered to mice via oral gavage for 7 days before the intraperitoneal injection of ethanol. Two hours after the injection of ethanol, liver tissues of each group of the mice were removed for the determination of catalase (**A**), glutathione peroxidase (GPx) (**B**), and manganese superoxide dismutase (MnSOD) (**C**) protein levels were determined by Western blot. Low-dose group (LD), 105 mg/kg coral hydrate; Medium-dose group (MD), 210 mg/kg coral hydrate; High-dose group (HD), 420 mg/kg coral hydrate; vehicle group (Veh), 420 mg/ kg coral calcium dissolved in 3% carboxymethyl cellulose. N = 6 mice for each group; * *p* < 0.05; ** *p* < 0.01; *** *p* < 0.001 as compared with vehicle group.

Since ethanol is known to damage the liver via oxidative stress, we also measured the anti-oxidative enzyme levels in liver tissue. We found that coral hydrate treatment significantly increased the protein levels of catalase (Figure 4A) and GPx (Figure 4B), but there were no significant changes in MnSOD when comparing coral hydrate and vehicle groups (Figure 4C).

### 3.5. Coral Hydrate Decreases Hepatic Inflammation

**Figure 5:**
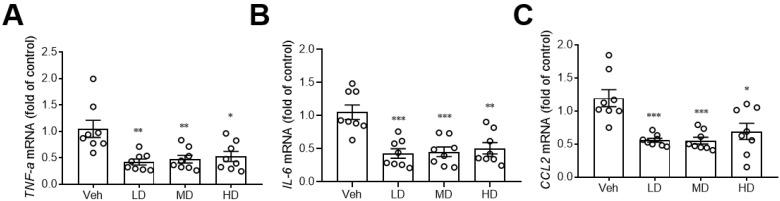
Coral hydrate attenuates ethanol-induced hepatic expression of inflammation markers. Coral hydrate was administered to mice via oral gavage for seven days before the intraperitoneal injection of ethanol for each group of the mice. Two hours after the injection of ethanol, liver tissues were removed for the determination of tumor necrosis factor-α (TNF-α) (**A**), interleukin-6 (IL-6) (**B**), and chemokine (C-C motif) ligand 2 (CCL2) (**C**) gene expression levels by RT-PCR. Low dose group (LD), 105 mg/kg coral hydrate; Medium dose group (MD), 210 mg/kg coral hydrate; High dose group (HD), 420 mg/kg coral hydrate; vehicle group (Veh), 420 mg/ kg coral calcium dissolved in 3% carboxymethyl cellulose. N = 6 mice for each group; * *p* < 0.05; ** *p* < 0.01; *** *p* < 0.001 as compared with vehicle group.

A previous study showed that hydrogenated water can reduce hepatic inflammation [16], so we also investigated the effects of coral hydrate on hepatic expression of inflammation markers. Notably, the coral hydrate treatments significantly decreased expression levels of pro-inflammatory cytokines, such as tumor necrosis factor-α (TNF-α; Figure 5A), interleukin-6 (IL-6; Figure 5B), and the chemokine (C-C motif) ligand 2 (CCL2; Figure 5C) in the livers of mice. These results implied that coral hydrate might be able to decrease ethanol-induced hepatic inflammation and further improve alcohol hangover.

### 3.6. Coral Hydrate May Affect Lipid Profile and Kidney Function in Mice

**Figure 6:**
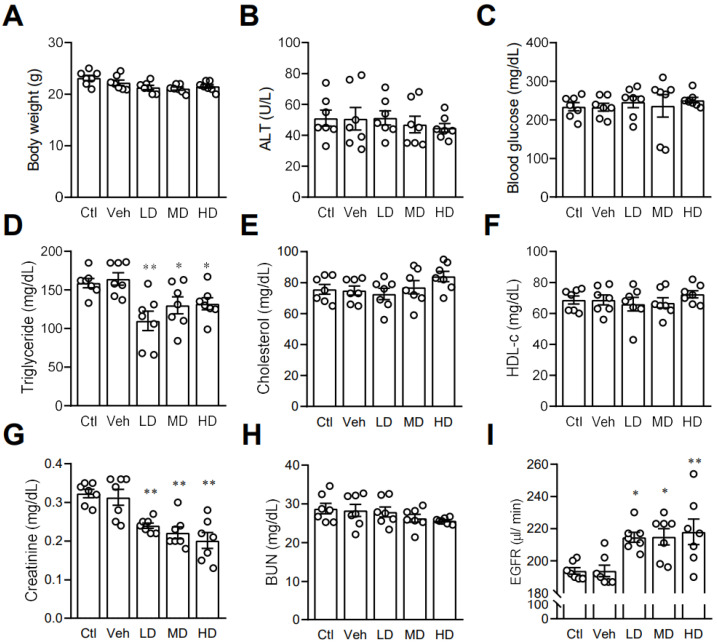
Coral hydrate affects lipid profile and kidney functions in mice. Coral hydrate was administered to mice via oral gavage for 7 days. After overnight fasting, the body weights (**A**) of the mice were recorded, and blood samples were collected for the determination of alanine transaminase (ALT) (**B**), blood glucose (**C**), and lipid profiles. Lipid profiles comprised triglycerides (**D**), total cholesterol (**E**), and high-density lipoprotein-cholesterol (HDL-c) (**F**). In addition, creatinine (**G**) and blood urea nitrogen (BUN) (**H**) were measured. Estimated glomerular filtration rate (EGFR) was calculated using chronic kidney disease epidemiology collaboration equation (**I**). All measurements were made with commercially available kits. Low-dose group (LD), 105 mg/kg coral hydrate; Medium-dose group (MD), 210 mg/kg coral hydrate; High-dose group (HD), 420 mg/kg coral hydrate; vehicle group (Veh) dissolved in 3% carboxymethyl cellulose. Control group (Ctl), 3% carboxymethyl cellulose. N = 7 mice for each group; * *p* < 0.05; ** *p* < 0.01 as compared with vehicle group.

After finding that coral hydrate had an effect on ethanol levels and catabolism enzymes, we next investigated its effects on other blood biochemistry indices. In order to assess the potential effects of coral calcium, we included a control group mice treated with 3% carboxymethyl cellulose. There were no significant differences between the control group and vehicle group. We found no significant effects of coral hydrate on body weight (Figure 6A), ALT (Figure 6B), or fasting blood glucose levels (Figure 6C). While administration of coral hydrate in mice did significantly decrease serum triglyceride concentrations (Figure 6D), total cholesterol (Figure 6E) and HDL-C showed no significant changes when comparing coral hydrate-treated groups to controls (Figure 6F). Notably, treatment of coral hydrate significantly decreased creatinine levels (Figure 6G) without changing BUN levels (Figure 6H). We also found the increased EGFR levels in mice treated with coral hydrate (Figure 6I). Thus, the coral hydrate treatment appears to influence multiple aspects of lipid profile and kidney function.

## 4. Discussion

In this study, we sought to determine the potential effects of coral hydrate on alcoholic intoxication or hangover using a mouse model. We first found that coral hydrate sustains its redox potential for a much longer time than hydrogenated water. Then, we showed that treatment of mice with coral hydrate significantly increased hepatic ADH and ALDH levels and clearance of alcohol and acetaldehyde, which corresponded to decreased sleeping time in ethanol-injected mice. In addition, coral hydrate increased the levels of hepatic anti-oxidative enzymes and decreased ethanol-induced hepatic inflammation. Furthermore, coral hydrate administration decreased serum creatinine and triglyceride levels in mice.

It has been previously reported that consumption of hydrogenated water can stimulate hepatic protective effects. For example, hydrogenated water protects against liver injury in choline-deficient, diet-induced nonalcoholic steatohepatitis mice [16]. In addition, intake of hydrogenated water decreased body mass and hepatic lipid accumulation in mice with high fat diet-induced nonalcoholic fatty liver [17]. Although numerous reports have detailed the beneficial effects of hydrogenated water in animals, the instability of the solution and overall bulk of the treatment limit its potential for clinical application. In this study, we found that porous coral materials in water can maintain redox potential for at least 24 h, potentially due to the stabilization of hydrogen molecules and sustained release of hydrogen into the solution. It is known that coral calcium is ionic and able to aid absorption and bioavailability of certain materials [18]. Since the gastric evacuation time in humans is approximately four hours after food intake, the sustained release characteristics of coral hydrate might increase its potential for application as compared with hydrogenated water. In addition to the advantageous sustained redox potential, coral hydrate intake can be easily quantified; this characteristic might also be beneficial in clinical settings.

Elimination of ethanol principally occurs via metabolism in the liver, with small amounts excreted in the urine and sweat [19]. An increase in hepatic ADH and catalase would be expected to facilitate the catabolism of ethanol, while improved kidney function may increase ethanol excretion. A previous study indicated that hydrogenated water alleviated ethanol-induced fatty liver in mice mainly due to its antioxidant activity [20]. In our study, we show that administration of coral hydrate not only increased anti-oxidative enzyme levels, but it also increased the levels of ADH and ALDH in the livers of mice. Upregulation of ADH and ALDH by coral hydrate should directly increase the rate of ethanol catabolism. In addition, the ROS-mediated induction of catalase is thought to facilitate the catabolism of ethanol [21,22,23]. In the present study, coral hydrate treatment increased the level of catalase, which could similarly enhance the catabolism of ethanol. Furthermore, we found that serum creatinine concentrations were significantly decreased after coral hydrate administration, which is suggestive of improved kidney function in mice. ROS plays a critical role in regulating nephron transport both via transcellular and paracellular pathways under physiological and pathological circumstances [24]. In addition, ROS affects kidney function and promotes damage in the kidney. In view of the anti-oxidative activity of hydrogenated water, a number of studies had been demonstrated that hydrogenated water has an activity to ameliorate kidney dysfunction [25,26]. With regard to the anti-oxidative activity of coral hydrate, we also observed the better kidney function in mice treated with coral hydrate. This effect might also play a role in enhancing ethanol catabolism.

In addition to improved kidney function, our blood biochemistry results revealed that mice treated with coral hydrate had decreased serum triglyceride concentrations. As redox modulation is known to play a crucial role in de novo lipogenesis [27], the anti-oxidative activity of coral hydrate might be related to the measured decreases in serum triglycerides. Alternatively, changes in the gut microbiome might mediate lipid metabolism [28] to produce the observed effects, since consumption of hydrogenated water alters gut microbiome and plasma metabolites in rats [29]. Thus, coral hydrate might not only directly regulate lipid metabolism in the liver, but it may also affect lipid metabolism indirectly through changes in the gut microbiome. Future studies will be needed to clarify the possible effects and mechanisms of coral hydrate on lipid metabolism.

There are still several limitations in the present study. First, although we found the hepatic ALDH expressions were increased, and the plasma acetaldehyde concentrations were significantly decreased in mice treated with coral hydrate after alcohol injection, the signs related to alcoholic hangover were not thoroughly confirmed since the animals were sacrificed after two hours of ethanol injection, and the further changes of the behaviour were not evaluated. In addition, gender difference could affect alcohol intolerance [30]. Estrogen modulates the ethanol-evoked myocardial oxidative stress and dysfunction [31] and it also plays a role in the increased vulnerability to ethanol-induced hippocampus plasticity impairment seen in females as compared with males [32]. In order to exclude the confounding effects of estrogen, we used male mice to evaluate the effects of coral hydrate on ethanol intoxication.

## 5. Conclusions

Our findings show that coral hydrate has sustained redox potential and exerts biological activities that can potentially improve alcoholic intoxication. The beneficial effects and characteristics of coral hydrate suggest that the reagent has excellent potential for application in clinical settings.

## Data Availability

The datasets generated during and/or analysed during the current study are available from the corresponding author on reasonable request.
